# Students Can (Mostly) Recognize Effective Learning, So Why Do They Not Do It?

**DOI:** 10.3390/jintelligence10040127

**Published:** 2022-12-16

**Authors:** Stephany Duany Rea, Lisi Wang, Katherine Muenks, Veronica X. Yan

**Affiliations:** Department of Educational Psychology, The University of Texas at Austin, Austin, TX 78712, USA

**Keywords:** learning strategies, college student, metacognition, expectancy value cost, motivation

## Abstract

Cognitive psychology research has emphasized that the strategies that are effective and efficient for fostering long-term retention (e.g., interleaved study, retrieval practice) are often not recognized as effective by students and are infrequently used. In the present studies, we use a mixed-methods approach and challenge the rhetoric that students are entirely unaware of effective learning strategies. We show that whether being asked to describe strategies used by poor-, average-, and high-performing students (Study 1) or being asked to judge vignettes of students using different strategies (Study 2), participants are generally readily able to identify effective strategies: they were able to recognize the efficacy of explanation, pretesting, interpolated retrieval practice, and even some interleaving. Despite their knowledge of these effective strategies, they were still unlikely to report using these strategies themselves. In Studies 2 and 3, we also explore the reasons why students might not use the strategies that they know are effective. Our findings suggest that interventions to improve learners’ strategy use might focus less on teaching them about what is effective and more on increasing self-efficacy, reducing the perceived costs, and establishing better habits.

## 1. Introduction

Being able to effectively regulate one’s study is important for student learning and performance at all levels of schooling (Nota et al. 2004; Ridley et al. 1992; Zimmerman 1986). As students progress through school—from elementary to (middle to) high school and into higher education—their need for self-regulation steadily increases, as students are expected to take more and more control over their own learning. In this world, where technological advances bring rapid changes, forcing people to learn new skills and adapt, knowing how to learn should be considered one of the most critical life skills. Moreover, with time being the ever-present limiting factor, learning must not only be effective but also be efficient. That is, just being motivated to put in a lot of study time is not enough; people must also know how best to use the limited time that they have.

However, do students know which strategies are most effective and efficient? In this introduction, we first review the strategies that research suggests are most effective and efficient and then examine the available evidence on what learners understand about these strategies. This literature suggests that students often lack knowledge of the effective strategies and that they often fail to incorporate these strategies into their own habits. Little is known about the barriers that prevent students from using these strategies. Thus, in the present studies, we investigate students’ awareness of effective learning strategies, their current study behaviors, and the reasons why they do not use effective learning strategies.

### 1.1. What Are Some Effective Strategies for Long-Term Learning?

Effective learning strategies are those that support meaningful knowledge construction and long-term retention, not just rote memorization and short-term performance (Mayer 2002; Soderstrom and Bjork 2015). These study strategies involve multiple components that make them so effective. First, learners must effortfully and elaborately process to-be-learned information, engaging in generative activities to select, organize, and integrate new information into broader knowledge networks (Fiorella and Mayer 2016). There are many strategies one could use to activate these processes. For example, testing oneself *before* learning (pretesting) has been shown to potentiate subsequent learning (Bjork and Bjork 2011; Kornell et al. 2009). These pretests are shown to lead to better learning even when learners take time out of studying the correct information to take a pretest in which they are almost guaranteed to be wrong (Kornell et al. 2009; Potts and Shanks 2014; Richland et al. 2009; Yan et al. 2014b). In other words, testing before studying is not only more effective than immediately jumping into studying, but it is more efficient too. Pretests are thought to promote learning by more efficiently directing attentional resources during study (Chan et al. 2018; Sana et al. 2021) and activating relevant semantic networks so that the new learning can be better integrated into prior knowledge (Carpenter 2017; Chan et al. 2018; Grimaldi and Karpicke 2012). The act of explaining concepts and phenomena, to oneself or to others, similarly engages deeper processing and leads to better long-term learning (Berry 1983; Chi et al. 1994; Schworm and Renkl 2006).

Second, learning does not happen in a single shot; rather, learners must repeatedly return to previously studied information. How these repetitions are sequenced matters: rather than cramming repetitions, spacing out repetitions (also known as distributed study or distributed practice) promotes better long-term retention (Carpenter 2017; Cepeda et al. 2006). In addition, learning is often augmented when the study of one concept is interleaved with similar, related concepts; this juxtaposition is posited to help learners distinguish between confusable concepts (Brunmair and Richter 2019; Carvalho and Goldstone 2017; Rohrer 2012). In empirical studies, spaced repetitions are compared with massed repetitions, and interleaved (i.e., mixed up) study is compared with blocked (i.e., one-at-a-time) study; in all comparisons, learners spend the same total amount of time studying, and it is merely the sequence of their study that differs. In other words, empirical studies provide evidence of both the effectiveness and the efficiency of both spacing and interleaving one’s study.

Third, for strengthening previously studied ideas and concepts, learners should not just reread but also practice retrieving the information from their long-term memories. The act of retrieval is thought to strengthen what is retrieved, making it more accessible in the future (Bjork 1994). Retrieval practice can take many forms. The most obvious strategy for engaging retrieval is to self-test, and many studies have shown the powerful pedagogical benefits of no-stakes or low-stakes quizzing (Agarwal et al. 2021; Yang et al. 2021). Retrieval can be engaged while explaining previously learned concepts to oneself or to others (as long as one is not relying on notes). It can be as simple as trying to write down everything one can remember on a blank piece of paper. In fact, studies have shown that this free recall technique leads to better long-term retention of information than spending the same amount of time rereading (e.g., Roediger and Karpicke 2006) or creating elaborative concept maps (e.g., Karpicke and Blunt 2011). Moreover, interpolating testing (interspersing segments of more-passive studying with tests) has been shown to discourage subsequent mind-wandering, promote better notetaking, and improve learning (Jing et al. 2016; Szpunar et al. 2013). In other words, students benefit from testing themselves before, throughout, and after study.

In contrast to these effective strategies (self-explanation, pretesting, spacing, interleaving, and retrieval practice), research has also shown that many commonly used strategies can be relatively ineffective for learning. These strategies are ineffective not because they cannot lead to learning gains but because these gains are either small or inconsistent, especially in comparison with other strategies. These include rereading, highlighting/underlining, and summarization (Dunlosky et al. 2013). Rereading is often used as the control condition in studies examining the benefits of generation, self-explanation, and retrieval (Rittle-Johnson 2006; Roediger and Karpicke 2006; Slamecka and Graf 1978). Although empirical evidence shows that people can learn more from a second reading, compared with the first reading, these gains are often small (Callender and McDaniel 2009; Raney 1993), and rereading is a much less efficient use of one’s study time compared with more-active strategies. Highlighting/underlining is considered relatively ineffective because learners often highlight/underline without engaging in much selection (Miyatsu et al. 2018). If summarization involves organizing and integration, it can be beneficial (Bretzing and Kulhavy 1979), but it is often categorized as a relatively ineffective strategy because it is often not done well, and it tends to be effective only for learners who are skilled at summarizing (Bednall and Kehoe 2011; Dunlosky et al. 2013).

An important distinction between the more effective and less effective strategies is how cognitively active they are. The more effective and efficient strategies, such as pretesting and retrieval practice, require learners to actively and effortfully process the to-be-learned content. These strategies are ones that lead all students to use more-active processes. Less-effective or -efficient strategies, including rereading, highlighting, and summarization, are mostly performed in passive ways that do not require students to engage in generative activities (Miyatsu et al. 2018). Of course, students *can* use more-active processes while engaged in these activities, but they often do not.

### 1.2. Metacognition and Self-Regulated Learning

Self-regulation is a multifaceted process, and many models have been proposed to describe the activities and aspects that comprise self-regulation (Panadero 2017). Contained within most models of self-regulation are metacognitive monitoring and control processes. That is, self-regulated learners reflect on and monitor their current state of learning (metacognitive monitoring) and make decisions about what they should focus on and how they should proceed (metacognitive control). Effective self-regulation of learning requires that students (a) accurately monitor their own learning (i.e., monitoring), (b) know what strategies are effective (i.e., knowledge), and (c) appropriately deploy these effective strategies (i.e., control).

#### 1.2.1. What Learners Understand about Effective Strategies: Metacognitive Monitoring and Control

In general, the existing cognitive literature has tended to portray learners as lacking the metacognitive knowledge of what strategies are effective for learning and the ability to accurately monitor their own learning, and hence, as making suboptimal self-regulated learning decisions (Bjork et al. 2013; Kornell and Bjork 2007; Lawson et al. 2019; McCabe 2011; Yan et al. 2016). Most empirical research shows that students often fail to accurately monitor the efficacy of different learning strategies (e.g., through the use of judgments on learning during experiments in which students experience learning using one or more strategies) and that they often choose to use suboptimal strategies in their own practice. For example, studies that manipulate spaced (versus massed) practice have found that students underestimate the power of spaced practice and often give higher judgments of learning to massed practice (e.g., Shaughnessy and Zechmeister 1992). Studies that manipulate retrieval practice (versus rereading) have found that students underestimate the benefits of retrieval practice and give higher judgments of learning to the rereading condition (e.g., Roediger and Karpicke 2006). Studies that manipulate interleaving (versus blocking) have found that students underestimate the power of interleaved study and give higher judgments of learning following blocked practice (e.g., Yan et al. 2016).

Surveys too have also found that students do not report using the most effective strategies. For example, surveys have highlighted that students tend not to return to previously studied course material (i.e., lack of spaced repetition) and underestimate the pedagogical benefits of testing (Kornell and Bjork 2007; Yan et al. 2014a). In another survey of undergraduate students, Karpicke and colleagues (2011) found that the most commonly reported study strategy was rereading, which is generally considered a less effective strategy.

#### 1.2.2. What Learners Understand about Effective Strategies: Metacognitive Knowledge

Failures of metacognitive monitoring and control do not necessarily imply that students lack the knowledge of effective strategies. They may have the knowledge but simply fail to put it into action. Blasiman et al. (2017) surveyed participants on a number of study strategies, asking them to report both how much they intended to use each strategy and how much they actually used each strategy. There were many strategies where there was a large discrepancy between the intended use and the actual use. The biggest discrepancies were for the use of flashcards and practice testing. This suggests that students’ knowledge of the benefits of retrieval practice outstrips their actual usage of it. On the other hand, the smallest intention–usage discrepancy was in rereading texts. To begin to address the ways we can improve students’ self-regulated learning, it is important to first understand what metacognitive knowledge they have, what metacognitive knowledge they do not have, and what barriers prevent them from using the strategies they know are effective.

##### Spacing and Interleaving

A substantial amount of research has focused on what students know about the benefits of spacing and interleaving. The bulk of this research highlights that students are very aware of the benefits of spacing. For example, Cohen et al. (2013) showed that participants will make a plan to spread out studying over more days if they anticipate having to hold on to that information for a longer amount of time (e.g., a week vs. a day). Susser and McCabe (2013) showed that the majority of participants could accurately identify that spacing out study in multiple shorter sessions leads to better long-term learning than massing that study in one longer session. Interleaving appears to be less well understood by students. Yan et al. (2016, 2017) showed that participants hold a priori beliefs that interleaving the study of related concepts is less effective for learning than blocking one concept at a time. This belief in blocking is resistant to direct instruction about the benefits of interleaving (Yan et al. 2016) and guides the way students construct real and hypothetical study schedules (Tauber et al. 2013; Yan et al. 2017). However, there are some nuances in students’ understanding: when allowed to choose a combination of blocking and interleaving, participants overwhelmingly choose a hybrid of the two over a purely blocked sequence (Yan et al. 2017). More impressively, open-ended responses revealed that 77% of participants wrote that they blocked in order to see similarities within a category, and 51% mentioned that they interleaved in order to see differences between the categories. These open-ended responses align well with the most dominant theory for when and why interleaving is beneficial for learning (Brunmair and Richter 2019; Carvalho and Goldstone 2017). Finally, an analysis of open data for Experiment 4 in Tauber et al. (2013) shows that while participants do choose to see several birds from the same family consecutively, they also often go back and forth between different bird families and revisit previously studied bird families multiple times.

##### Retrieval Practice

In Karpicke et al. (2009), the fact that rereading was the most commonly reported strategy is often interpreted as students’ lacking knowledge of effective studying. However, the second most commonly reported study strategy was self-testing. Given that self-testing and rereading often go together (self-testing is more effective and can increase motivation to studying when followed by feedback; Abel and Bäuml 2020), it is possible that the students have a greater understanding of the benefits of retrieval practice than they are given credit for. Blasiman et al. (2017) also presented some mixed data: participants reported relatively high intentions of using flashcards and practice tests (ranked second and fourth out of ten strategies in terms of intended use). However, while using flashcards was rated as one of the more effective strategies, practice testing was rated as one of the less effective strategies (and reading notes was rated as the most intended, most used, and most effective strategy).

What is unclear from the existing data is what students understand about sequencing reading and testing. Pretesting potentiates future study (Pan and Sana 2021), interpolated tests improve subsequent focus and the integration of knowledge (Jing et al. 2016), and retrieval practice promotes long-term retention (Yang et al. 2021). In other words, these lines of research imply that students should be testing themselves throughout their study, interpolating tests with rereading (i.e., test themselves after chunks of learning, rather than only after everything at once). However, do students appreciate the benefits of pretesting and testing themselves throughout the process of studying, or do they test themselves only at the end of studying as a way of checking what they do or do not yet know? How would they choose to balance their time between rereading and testing?

##### A Mixed-Methods Approach

One limitation of many of the existing studies is that only the strategies that researchers have included as part of their surveys are measured (but see Karpicke et al. 2009). Cognitive psychology researchers have tended to be most interested in spacing, interleaving, and retrieval practice. Some studies have included other strategies too (e.g., Blasiman et al. 2017), but even these expanded surveys tend to leave little room for participants to offer their own, uninfluenced ideas of what effective studying looks like. For example, students might report the usage of similar studying strategies (e.g., reviewing, rereading, quizzing, etc.) but have a very different approach to these strategies (e.g., passive vs. active review, term-definition vs. conceptual flashcards, or creating their own quiz questions vs. relying on pre-existing materials). A handful of studies have examined open-ended responses from students, revealing insights that other close-ended surveys have not (Karpicke et al. 2009; Tullis and Maddox 2020; Zepeda and Nokes-Malach 2021). For example, Tullis and Maddox (2020) found that middle and high school students differed in the reasons why they use retrieval practice (significantly more high school students reported using it as a metacognitive tool, checking what they do or do not know). Hence, in our present studies, we use a mixed-method approach to clarify participants’ responses.

#### 1.2.3. Why Do Students Not Use the Strategies They Know Are Effective?

Susser and McCabe (2013) found that participants are more likely to space than mass in preparation for an upcoming test when there is a lot of material to learn, the material is perceived as more difficult or more valuable, the test is weighed more heavily, and there are fewer commitments in the week of the test. In Biwer et al. (2020), participants were given an intervention in which they learned about various effective strategies; yet participants did not always use the strategies they learned. Focus group interviews of a subset of these participants revealed that although students wanted to use retrieval practice, they often believed that it took too much time (especially if there was a lot of material to cover).

This work is consistent with theories on motivation, including situated expectancy-value theory, which posits that students’ motivation to engage in a task is a function of their expectancy or beliefs about whether they can succeed at the task and their value of the task (i.e., how interesting, important, and useful they perceive the task to be). However, this theory also posits that students will be *less* motivated to engage in tasks in which they perceive high costs (e.g., will take too much time/effort or will lead to more stress/anxiety; Eccles and Wigfield 2020). In other words, contextual and motivational factors appear to be important, and yet these have not been systematically studied with respect to students’ engaging in effective learning and studying strategies. Understanding the challenges that students face while trying to incorporate these strategies is much needed in this field of research.

### 1.3. The Present Studies

Our review of the literature yields several gaps. First, there are gaps in understanding what students know about more-effective and less-effective strategies for learning. In Studies 1 and 2, we asked participants about hypothetical or imagined students, rather than to describe their own intended or actual study behaviors (which may be a combination of beliefs about the effectiveness of different strategies and other factors, such as perceived costs). Furthermore, asking participants to describe hypothetical others creates a psychological distance and reduces socially desirable responses to better determiner what people truly believe (Constant et al. 1994; Evans et al. 2015; Hughes 1998). To determine the metacognitive knowledge of both effective and less-effective strategies in Study 1, we asked participants to describe a lower-achieving (bottom 10%) student, an average student, or a higher-achieving (top 10%) student. Participants were asked to give open-ended responses in order to generate strategies that would not be influenced by experimenter-designed questions. To address the need to better understand what students know about the benefits of retrieval practice, we created a question that would obtain more fine-grained detail to understand how participants use testing in conjunction with rereading. Finally, to address the need to better understand how students balance their time between the more and less effective strategies, we presented questions that directly pitted pairs of strategies against each other.

Second, there are gaps in understanding the perceived barriers that prevent students from using these strategies. In Study 2, we created three vignettes of students’ using different sets of study strategies (passive, metacognitive, active). After establishing what participants believed about the efficacy of each set of study strategies, we asked participants about how much of their own study strategies resembled each and the reasons why they might not use each set of study strategies. This allowed us to examine and compare the perceived barriers for each of the different sets of study strategies—the more and the less effective ones. In Study 3, we focused on the barriers of use for effective strategies. Using a group of students who had received direct instruction about effective strategies (elaboration, spacing, interleaving, pretesting, and retrieval practice), we tracked their usage and perceived barriers across an academic semester. In Studies 2 and 3, we generated a list of barriers that were informed by motivational theory. Following situated expectancy-value theory (Eccles and Wigfield 1995, 2020), we asked about students’ expectancy (e.g., whether the student believed that they could use the strategy effectively), value (e.g., whether the strategy would be useful or effective), and cost (time, effort, psychological; Flake et al. 2015; Jiang et al. 2018) of using effective studying strategies.

## 2. Study 1

In Study 1, we examined whether participants would describe high-achieving, average, and low-achieving students using different types of strategies, through a combination of open-ended and closed-ended questions. The differences in these responses provide information on what students understand as being more and less effective for learning.

### 2.1. Methods

#### 2.1.1. Participants and Design

Participants were 300 students (49% women, 48% men, 3% nonbinary people; mean age = 21.24, *SD* = 1.99, range = 18–30; 60% White, 22% Asian, 15% Hispanic or Latinx, 12% Black or African American, 4% Native Hawaiian or Pacific Islander, 4% Native American or Alaska Native, and 1 participant preferred not to answer) recruited in March 2022 from Prolific (www.prolific.co), a survey-based website that is widely used for research. Using the Prolific screeners, we restricted our participants to self-identified undergraduate students living in the United States, between 18 and 30 years of age. We aimed to collect 100 participants per condition because that is the sample size sufficient to detect medium effect sizes (*d* = 0.40) between two independent groups at α = 0.05 with 80% power (G*Power 3.1; Faul et al. 2007).

Participants were randomly assigned to one of three conditions: top-10% student, average student, and bottom-10% student. The manipulation was a between-participants design because the goal was to collect detailed open-ended responses; a within-participants design would have resulted in a long survey, and we did not want participants to become fatigued.

#### 2.1.2. Materials and Procedure

The study was administered fully online, and the full survey is presented in Supplemental Materials. First, after providing consent, participants were randomly assigned to bring to mind one of three types of students: a bottom-10% student (*n* = 105), an average student (*n* = 102), or a top-10% student (*n* = 92). The following prompt was presented: “Think about a student who is a(n) [top-10%/average/bottom-10%] student. This student is currently enrolled in college and is a STEM major. Bring the image of that student to mind. Who is this student? What do they do in and out of class? Maybe this is someone you know or maybe it is just someone you made up in your head. Do you have a good image of this [top-10%/average/bottom-10%] student in your mind? Proceed to the next page once you have a good image of this [top-10%/average/bottom-10%] student.” This prompt was designed to encourage students to think more deeply about the imagined student before moving on to the more specific questions; students did not type any response to this prompt.

Participants were then asked both about the imagined student’s responses to struggle and the imagined student’s learning strategies. Whether they answered questions about the learning strategies first or second was randomized among participants. We also asked them about the imagined students’ motivation but did not describe the motivation items further, as they are not central to our main research question^1^, but they are detailed in the Supplemental Materials.

##### Experiences of Struggle

Participants were given a scenario: “This [top-10%/average/bottom-10%] student is finding it really hard to understand one of the core concepts in their course. What might be the reasons as to why they are struggling?” Participants first wrote an open-ended response to this question, and then on the next page, they selected from a list of options what they thought was the most likely reason. They were then asked what the imagined student should do in this situation, where they are struggling. Again, they first typed in their own response in a textbox and then selected from a list of options what they thought was the most likely thing the imagined student would do when struggling. Finally, they answered an open-ended question: “When this [top-10%/average/bottom-10%] student is in the process of learning, what should it feel like?”

##### Learning Strategies

We examined the learning strategies that participants associated with each imagined student within a variety of questions, described below. Specifically, we asked them to imagine that this student is enrolled in a course that has a cumulative final exam. Our questions were about how this imagined student would study for this final. First, to encourage students to be more specific in imagining how this student would study, we asked two questions about when they would start studying and where that imagined student would study (the location or locations and whether that imagined student would be studying alone or with friends). With this context established, we then turned to our questions of interest: the learning strategies.

First, we asked about the frequency of using various strategies. Participants were asked to rate on a 5-point Likert scale (1 = never—they never do this; 5 = very frequently—they do this almost every time) how often the imagined student would engage in five types of learning strategies: reread, highlight or underline, make notes, test themselves, and explain concepts to themselves or others. For each strategy, participants were presented with a list of examples of what that strategy might look like (e.g., for testing themselves, the examples were to either take practice tests, use flashcards, or recall already-learned things from memory without peeking at their notes).

Next, to obtain more detail on the ways participants imagined how these students would test themselves, we asked a follow-up question that garnered more detail on retrieval practice. Specifically, we created four multiple-choice options that represented two dimensions of test use: timing relative to rereading (review first and then testing or testing both before and after review—to gauge participants’ understanding of how the three types of students would differently engage in pretesting) and chunking of information (whether content is split into chunks—to gauge their understanding of how the students would differently engage in interpolated testing).

Finally, we asked three questions that required participants to compare pairs of strategies: listening to explanations versus explaining to self or others; rereading versus testing; and focusing on one concept/skill (blocking) versus reviewing a mixture of concepts/skills (interleaving). For each question, we asked participants to indicate the proportion of time spent on each activity within the pair.

##### Demographics

Finally, participants completed a demographics questionnaire (age, gender, race/ethnicity, year in college, college major, college GPA, and first-generation college student status).

### 2.2. Results

#### 2.2.1. Imagined Students Differ in Reasons for and Responses to Struggle

Participants were asked, what are the likely reasons that their imagined student was struggling (summarized in Table 1) and what was that imagined student likely to do when they found themselves struggling (summarized in Table 2)? We were most interested in how likely participants were to focus on the use of learning strategies. The use of ineffective strategies was the most likely reason participants gave for the average student (32% of responses) and the second most likely reason (25% of responses) participants gave for the bottom-10% student, suggesting that participants do believe that strategies are important and can make a difference. Using ineffective strategies was less likely to be reported to be the reason for the top-10% students (17% of responses), potentially because they think that those students would already be using more-effective strategies. Interestingly, despite the use of ineffective strategies’ being one of the top reasons for struggle, “change study strategy” was rarely what participants thought the imagined students would do (7–9% of responses)—perhaps they do not know how they should be changing their strategies. Instead, responses to struggle were mostly about seeking help (40–55%); seeking help might involve asking others for better study strategies, but we did not do any further probing.

#### 2.2.2. Imagined Students Differ in Frequency of Learning Strategies

**Figure 1:**
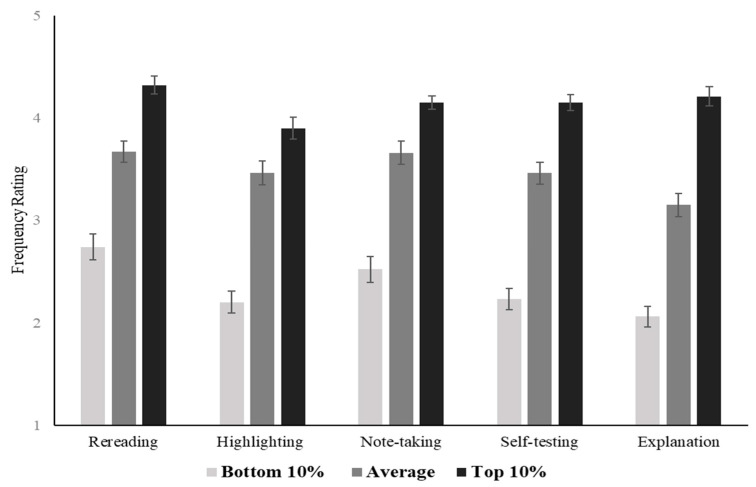
Quantity of strategy use: frequency rating of strategy use by condition. Error bars represent one standard error.

The first set of questions about learning strategies focused on the quantity, not necessarily the quality, of strategy use. They were presented with a list of strategies and asked to rate how often they used each. Some of the strategies were considered relatively passive (rereading, highlighting, and underlining), and some were considered relatively more active (notetaking, testing, and explanation). Figure 1 shows the mean ratings for each strategy by condition. Participants described the higher-achieving students as using all strategies more frequently. One-way ANOVA tests were conducted for each learning strategy and confirmed that there were significant differences in frequency ratings between conditions for all strategies: *p*s < 0.001. Post hoc Tukey’s HSD tests found that all pairwise comparisons were significantly different: *ps* < 0.01, Cohen’s *d*s = 0.40–2.24. The full descriptive statistics and one-way ANOVA results are presented in the Supplemental Materials (Table S1).

#### 2.2.3. Imagined Students Differ in the Quality of Learning Strategies

The next set of questions focused on the quality of strategies used.

##### Use of Testing

Participants were asked a question that more specifically examined how the imagined students would use self-testing. They were presented with four items and asked to select which one most likely resembled the testing habits of their imagined student. These four items represented two dimensions. Two of the items reflected dividing the to-be-studied information into chunks: the better the imagined student, the more likely they were to be described as chunking the information. Two of the items reflected testing throughout the whole study process rather than only after reviewing: the better the imagined student, the more likely they were to be described as testing themselves both before and after reviewing. The responses are presented in Table 3. Chi-squared goodness-of-fit tests for each dimension revealed significant differences between the conditions (*χ*^2^(2) = 31.44, *p* < 0.001) for the chunking dimension and the conditions (*χ*^2^(2) = 44.79, *p* < 0.001) for the testing order dimension. Post hoc chi-squared goodness-of-fit tests revealed that all conditions were significantly different from each other: *p*s < 0.022 (see summary in Supplemental Materials, Table S2).

##### Comparison of Pairs of Learning Strategies

**Figure 2:**
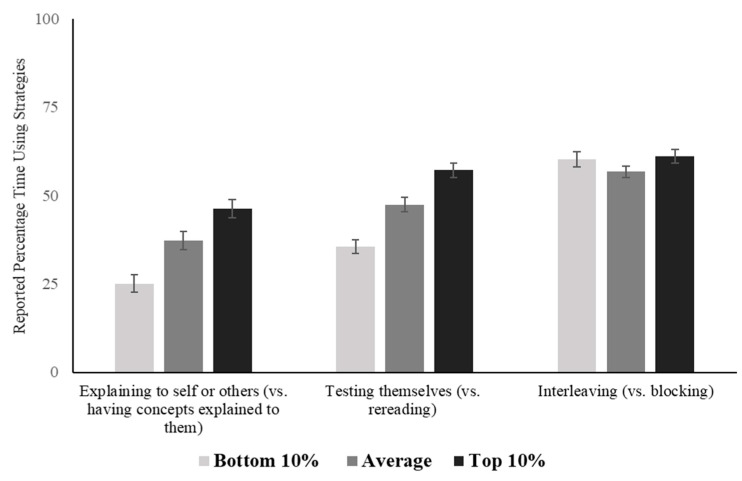
Quality of study: reported percentage of time using the more (vs. less) effective strategy, by condition. Error bars represent one standard error.

Participants were presented with pairs of strategies and asked how their imagined student divided their study time between each item in the pair. Each pair consisted of a more passive, less effective strategy and a more active, more effective strategy. For ease of interpretation, the responses were coded such that higher numbers reflect more use of the active strategy (explaining, testing, interleaving). The average responses by imagined student conditions are presented in Figure 2. There were no differences between the conditions in how often the imagined student used interleaving (vs. blocking): *F*(2, 297) = 1.04, *p* = 0.354. There were differences, however, in the other two pairs, and the higher-achieving students were described as using the more effective strategies. One-way ANOVAs for each strategy pair (with the exception of blocking versus interleaving) confirmed that the three conditions differed significantly, and post hoc Tukey’s HSD tests showed that the differences were significant between all three conditions: *p*s < 0.01, Cohen’s *d*s = 0.45–0.98 (see Supplemental Materials Table S3 for details).

In other words, participants appear to understand that better learning and achievement are related to increased use of self-explanation and retrieval practice. However, the use of interleaving (versus blocking) was not perceived to be related to better learning and achievement. Interestingly, the overall rates of the predicted usage of interleaving were high relative to self-explanation and retrieval practices, suggesting that interleaving may be considered a relatively frequent strategy in general.

##### Qualitative Analysis of Open-Ended Descriptions of Study Strategies

We also asked participants to write what strategies these imagined students would be using when studying for a cumulative exam for a course. In coding these responses, two coders (SDR, VXY) read through a random selection of the first 50 responses together and generated a list of categories. At this first pass, the priority was to generate a diverse number of codes that could well capture the variation in the responses. Next, through discussion, they grouped similar codes (e.g., “use flashcards” and “test self-using Quizlet” were combined; “reviewing notes” and “reread notes” were combined). This process resulted in a final list of 15 codes. The remaining responses were coded by SDR and an undergraduate research assistant. These two coders coded 20 responses together and then split the remaining responses between them. SDR and VXY had a final discussion, and all responses that had been coded by only one person were then checked by either SDR or VXY. In this way, every response was coded by at least two people. All coding was conducted without looking at the assigned condition of the participant.

Table 4 summarizes the frequency with which different categories of learning behaviors were found within the open-ended descriptions in each description. We also coded the responses as strategies that tend to be more active or more passive. Overall, the majority of the responses for the bottom-10% student involved either no strategies or very passive strategies: skimming through notes, cramming, and re-reading. As we expected, the average student was associated with a mix of passive and more-active strategies, such as flashcards, reviews, and practice problem exercises. The top-10% student was associated with more-active strategies compared with the other two conditions, as many of the participants reported the use of actively reviewing notes, practice quizzes, reviewing previous assignments, and actively solving problems from online and textbook resources as a combination.

Regardless of whether a strategy tends to involve more- or less-active processing, we also noticed how the described use of each strategy differed within each category. That is, even when participants were reporting the same strategies, the quality of their descriptions differed by condition. Table 5 illustrates some of these differences. For instance, when participants said that their imagined student would “review notes,” the way this was described often differed by condition. For the bottom-10% student, reviewing notes might be described as briefly reviewing notes that were often not their own. For the average student, reviewing notes might be described more actively, such as looking at their notes and past homework. For the top-10% student, reviewing notes might be described in even-more-active and metacognitive ways, such as using more concentration during review and paying attention to what they do not know.

### 2.3. Discussion

This study demonstrates that undergraduate participants can readily distinguish between more- and less-effective strategies. Participants understand higher-achieving students as putting in more study time and using all types of strategies (including the more active ones, such as self-testing, explanation, and notetaking). However, not only quantity mattered for higher-achieving students; participants also described these higher-achieving students as using that study time in qualitatively different ways. Participants reported that the highest-achieving students would engage in more-spaced and -interpolated retrieval practice (e.g., chunking, testing before and after), more self-testing compared with rereading, and more self-explanations than listening to explanations, compared with the average and low-achieving students. However, the conditions did not differ in how much they described the use of interleaving, suggesting that the benefits of interleaving are not as well understood. Open-ended coding also revealed qualitative differences between the conditions. For example, the use of notes is a particularly interesting one to consider. Past studies have shown that students overwhelmingly engage in notetaking but that they often use it as a way to pay attention or as external storage (Morehead et al. 2019; Witherby and Tauber 2019); our qualitative analyses show that learners are sensitive to how notes can be used more or less effectively.

**Table 5:** Example Responses for Commonly Reported Strategies.

Strategy Code	Bottom 10%	Average	Top 10%
Reviewing notes	They would briefly look over the information	Reviewing notes, looking through PowerPoints, and looking at any external resources	They would review the material thoroughly and then ask questions on the material to other students and teachers.
Practice problems	They would self-practice the same exam repeatedly until they feel confident	The student would take notes while reviewing the content and then do practice problems to reinforce the knowledge.	They will try to practice problems and revise homework, especially where they made mistakes in their homework.
Flashcards and/or Self-testing	They would use flashcards and try to memorize their notes by looking at them.	I would expect a lot of flashcards (Quizlet) and practice exams would be the best way to ensure success.	I think they would use active recall to test themselves on the material, such as flashcards with questions.
Making flashcards	They may make flashcards with the terms on one side and the definitions on the other.	This student would make notecards to study from throughout the day.	Make notes throughout the semester. Ask about what topics will be on the final. Make flashcards of final exam material.
Study with friends or classmates	Study all the class lectures, look up questions online, and maybe ask a friend.	They would probably try to link up with other students to exchange notes and go over the material together.	The student might participate in study groups where their classmates collaborate and quiz one another. They will likely review their notes. They might make flashcards.
Seek help	By going to office hours and tutoring sessions	The student would also most likely go to office hours to ask questions to the professor and/or teaching assistants.	They would discuss the material with teaching assistants or tutors to ensure they understood it.
Use online resources	They would look at the little notes they have and google some things they think would be related to the class.	Watch examples being done on YouTube.	See if there are any resources online for practice problems, go over the concepts they have struggled the most with, and devote the most time to those.

## 3. Study 2

Whereas Study 1 had a between-participant manipulation, Study 2 had a within-participant manipulation. In Study 2, we presented three vignettes of students studying in different ways: one student puts time and effort into using active strategies (e.g., spacing, self-testing, concept mapping, elaboration); another uses relatively passive strategies (e.g., rereading for hours, highlighting important sections); and a third uses metacognitive strategies (e.g., planning, assessing gaps, goal setting, help-seeking). We wanted to investigate how participants differentiated and related these types of strategies to academic outcomes (i.e., performance and learning). Study 2 also asked participants about the potential reasons why using the strategies shown in the three vignettes would not be their own strategy choice.

### 3.1. Methods

#### 3.1.1. Participants and Design

Participants were 517 undergraduate students (62% women, 37% men, and 1% nonbinary person; mean age = 20.49, SD = 2.1, age range = 18–37; 38% White, 26% Asian, 23% Hispanic or Latinx, 7% Black, 2% Middle Eastern, 2% Asian American, 1% Native American or Alaska Native, 1% Native Hawaiian/Pacific Islander, and 1% other), recruited from an undergraduate participant pool at the University of Texas at Austin. The study was conducted fully online and participants were compensated with partial course credit. Participants read and rated all three vignettes (i.e., within-participants design). There was no a priori power analysis to determine the sample size; we simply left the study open in the institutional participant pool until it closed. A post hoc sensitivity analysis using G*Power 3.1 (Faul et al. 2007) revealed that the study would be able to detect effect sizes as small as *f* = 0.06 (i.e., a small effect) at α = 0.05 with 80% power.

#### 3.1.2. Materials

The key portion, the vignettes, of the survey, are described below, but the survey also contained several other measures that were not of central interest (e.g., motivation-related beliefs and information on currently enrolled courses). The full set of materials, with the exact language shown to the participants, is detailed in the Supplemental Online Materials (Table S4).

The participants were presented with three vignettes describing different students. These students were presented to participants as Student A, Student B, and Student C, and the types of strategies that each student in the vignettes had were boldfaced. The vignette for Student A represented an active student:

“Student A studies for her exams by trying to **think deeply** about the material that she has learned. She tries to **space out her studying** over the course of a few days or weeks before her exam. During each study session, she tries to **quiz herself** on material she has learned, and she tries to **elaborate on each topic**, using techniques like **mapping out how different concepts relate to one another.**”

Student B represented a passive student who used more-passive strategies (e.g., carefully reviewing the material, studying for hours at a time, focusing on important facts, and highlighting or underlining passages). Student C represented a metacognitive student who used more metacognitive strategies (e.g., planning their studying, focusing time and effort, using techniques such as goal setting, and seeking out help). For each question, vignettes were always presented in the same order: Student A (active), Student B (passive), and Student C (metacognitive).

#### 3.1.3. Procedure

The study was administered online. After providing informed consent, participants were told that they would be presented with descriptions of three students who are studying for an exam and that researchers wanted to know what they thought of these students’ approaches to studying. The three vignettes were then presented on the same page, but each one was presented in a different color to aid differentiation. Participants were required to spend at least 20 s reading this page.

##### Rating Vignettes

Next, they were shown one vignette at a time and asked questions about each one. Everyone saw the same order: Student A, Student B, and Student C. The “active,” “passive,” and “metacognitive” labels were never used. For each vignette, participants rated what they thought would be the performance and learning level of the student in the vignette on a 7-point Likert scale (1 = not true at all, 7 = very true). Two questions pertained to performance: Student [A/B/C] is likely to do well on their exam; Student [A/B/C] will do well in their classes if this is how they study. Two questions pertained to learning: Student [A/B/C] will learn a lot during their study time; Student [A/B/C] will be likely to remember this material a year from now.

##### Comparing Vignettes

After participants rated each vignette, they were then presented with the three vignettes on the same page again (so that they would not have to rely on their memories), and then they were asked to compare the three students and choose who they thought would do best on the exam, uses the most effective strategies, learns the most during their study time, and is most likely to remember the material a year from now.

##### Similarity of Own Studying to Vignettes

Next, participants were asked to think about how they study for the class they cared the most about this semester. They were then shown each vignette, one at a time. For each one, they were asked, “how often do you use strategies similar to Student [A/B/C] when you study for [the class they care the most about]?” They responded to this question on a 5-point scale: 1 = never, 5 = very often. Then, they were presented with a list of possible reasons why they might not use the strategies from the vignette. The list of reasons included two items each that covered metacognitive knowledge (e.g., this way of studying will not help me on my exam) and self-efficacy (e.g., I do not know how to study in this way), as well as one item each about time cost (e.g., I do not have time to study in this way), effort cost (e.g., I do not want to put in the effort to study in this way), and three subtypes of psychological cost (i.e., nervous, boring, difficult; e.g., this way of studying makes me feel nervous, worried, or anxious). They were asked which were the main reasons why they did not use those strategies and were allowed to check all that applied. After they made their choices, they were presented with only the options that they selected and asked to choose the number one reason for not using these strategies. They repeated this procedure for each of the three vignettes.

Next, they were asked to think about how they study for the class they care the least about this semester. They were then shown all three vignettes again and asked to rate how often they use strategies similar to each student on a scale of 1 (never) to 5 (very often).

##### Demographics

Finally, participants were asked about their demographics (age, gender, ethnicity).

### 3.2. Results

#### 3.2.1. Participants Identify Active Strategies as Most Effective for Learning and Performance

**Figure 3:**
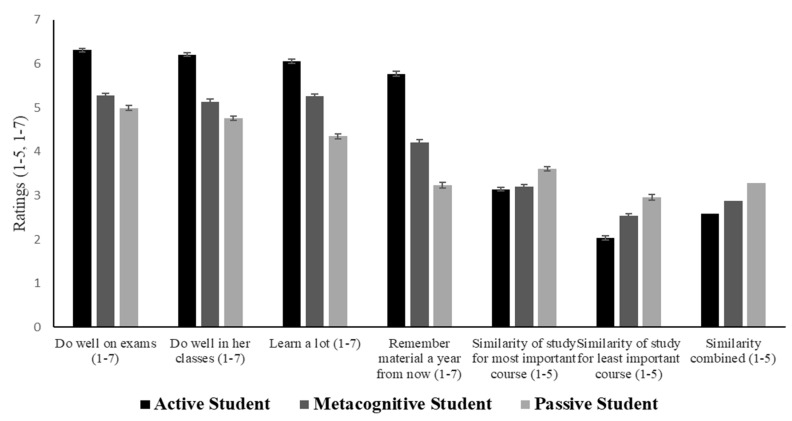
Means performance, learning, and similarity to own behaviors ratings, by vignette. Error bars represent one standard error.

Participants were asked to rate the learning and performance that would be expected of each described student. These average ratings, by condition, are presented in Figure 3. A one-way multivariate analysis of variance (MANOVA) was conducted to examine whether there were significant differences between the three vignettes for each of the four ratings. The MANOVA showed that there were significant differences for each rating: *p*s < 0.001 (see Supplemental Materials Table S5 for detailed MANOVA results). Post hoc *t*-tests revealed that all pairwise comparisons were significantly different, *p* < 0.001. The effect sizes between the active and passive learners ranged from Cohen’s *d*s = 1.21–1.83; the effect sizes between the active and metacognitive learners ranged from Cohen’s *d*s = 0.66–1.12; the effect sizes between the metacognitive and passive learners ranged from Cohen’s *d*s = 0.25–0.68 (see Supplemental Materials Table S6 for full descriptive and analytical results).

Participants were presented with four statements about learning and performance and asked to make categorical judgments about which of the three students best fit each statement. Table 6 shows how often each student was selected in response to each statement. Student A was overwhelmingly selected more often than the other two students; chi-squared goodness-of-fit tests confirmed significant differences.

#### 3.2.2. Participants Report That Their Own Study Behaviors Most Resemble Passive Strategies

The similarity ratings of each student vignette to participants’ own study behaviors for their most-cared-about class and least-cared-about class are presented in the right-hand side of Figure 3. A two-way repeated-measures ANOVA revealed a main effect of class type: participants gave higher similarity ratings when thinking about how they study for the class they care about the most than the class they care about the least: *F*(1, 3090) = 66.18, MSE = 87.39, and *p* < 0.001, *η_p_*^2^ = 0.02. There was also a main effect of study strategy type: *F*(2, 3090) = 12.37, MSE = 16.33, *p* < 0.001, and *η_p_*^2^ = 0.008. Despite understanding which strategies were most effective, participants reported studying more similarly to the passive student (*M* = 3.28, *SD* = 1.00) than to either the metacognitive student (*M* = 2.87, *SD* = 0.95) or the active student (*M* = 2.59, *SD* = 0.81); post hoc *t*-tests revealed that all three were significantly different from each other: *p*s < 0.001 (see Supplemental Materials Tables S7 and S8 for the full results). There was a significant interaction between strategy condition and class: *F*(2, 3090) = 1.320, MSE = 4.00, *p* = 0.048, *η_p_*^2^ = 0.002. For the least-cared-about class, there were significant differences between all three strategy types: *p*s < 0.001. For the most-cared-about class, the frequency with which active strategies were reported was somewhat higher: it matched that of the metacognitive strategies, though it remained lower than that of the passive strategies.

#### 3.2.3. Barriers to the Use of Different Types of Strategies

**Figure 4:**
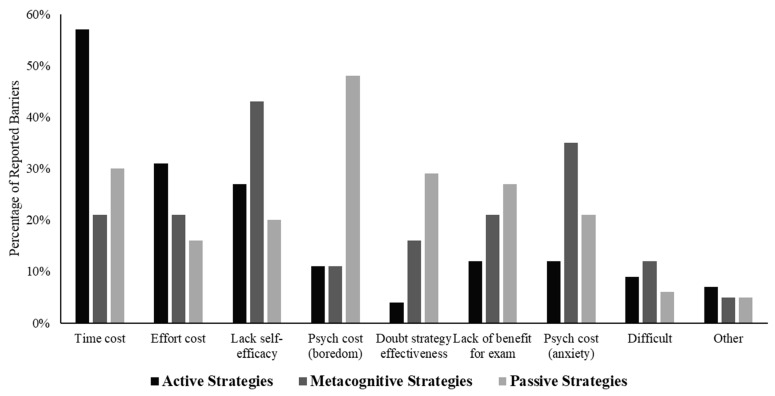
Percentage of reported barriers per vignette.

The percentage of participants who selected each barrier for each vignette is presented in Figure 4. The reported barriers differed for each type of study strategy. The most commonly reported barriers to using the active strategies were that these strategies took too much time (57%), these strategies required too much effort (31%), or the students did not think they could use these strategies effectively (27%). In other words, students expressed concerns about self-efficacy and cost. For the metacognitive strategies, self-efficacy was also a common concern (43%) and so was cost, but instead of time and effort, the primary concern was a psychological cost—that the strategies would make them feel anxious or nervous (35%). In contrast, the most commonly reported barriers to using passive strategies were that participants reported finding their approach boring (48%), these strategies took too much time (30%), and these strategies were not effective—that they would not learn (29%) and that strategies would not help them prepare for their exams (27%).

### 3.3. Discussion

Echoing the results of Study 1, Study 2 showed that participants can recognize more-effective (active) strategies from less-effective (passive) strategies. Yet their own study habits are more likely to resemble those of the less effective strategies, supporting previous findings (Karpicke et al. 2009). It did not matter whether participants were thinking about their most- or least-cared-about class; they still reported using more-similar strategies to those of the passive student (e.g., reviews, highlighting, and long study sessions) than of the active or metacognitive students. The study further highlighted the motivational barriers that students report to using different sets of strategies.

## 4. Study 3

In Study 3, we surveyed students who had been directly taught about effective strategies, to make sure that metacognitive knowledge was not a key variable in how students reported their barriers to strategy use. We tracked how the perceived barriers changed throughout the semester.

### 4.1. Method

#### 4.1.1. Participants and Design

We analyzed archival data that had been collected from 95 students across two sections (41 in one section and 54 in the other) of an undergraduate course titled “*Cognition, Human Learning, and Motivation*” at the University of Texas at Austin in the fall of 2019. A pre-semester survey was sent out to the students to learn more about them; 106 students responded to this survey. There were some changes in the student roster during the drop/add period that followed, so these survey data are described purely to provide additional context about the course. Roughly one-quarter of the students were first-year students, half were second-year students, and the remaining were about equally split between third- and fourth-year (or higher) students. The majority of students were enrolled in a college of education major or teacher training (78%). Most students reported that they had some plan to teach in the future, 59% planning to teach in elementary school, 4% in middle school, and 18% in high school. Some were unsure, and only 12% said that they had no plan to teach in the future.

In this course, students were taught about “desirably difficult” strategies—pretesting, generation, spacing, interleaving, and retrieval practice. These strategies were taught in Week 3 of the course and repeatedly reiterated throughout the semester. Initial instruction involved being shown evidence from empirical studies, as well as small-group and whole-class discussions on the reasons why these strategies are effective. Attention was also explicitly and repeatedly drawn to the aspects of the course that used these learning principles: students took a pretest on to-be-lectured content (due the day before the lecture), each lecture started with a brief retrieval practice of the main takeaways from the previous week’s classes, lectures were interactive (often requiring students to generate their own ideas before being taught the correct concepts), and weekly quizzes were cumulative (i.e., spaced and interleaved retrieval practice). Finally, the course content and, in particular, these learning strategies were framed as having high utility value for students’ own lives—connections were drawn to the benefits of these strategies for mastery goals and performance goals (better retention for exams and reduced anxiety and stress when preparing for exams).

In other words, these were students who presumably had the metacognitive knowledge of what strategies are effective and the motivation to learn about learning. There were three unit exams (held on Weeks 5, 9, and 13) and a final exam (on Week 15).

#### 4.1.2. Materials and Procedure

The undergraduate students received printed exam packets. The bonus questions were printed on the last page at the end of each exam packet (see Supplemental Materials for the full set of questions). All the bonus questions for all the exams were presented in the same order and format. The bonus questions were completely voluntary; students were given one bonus point for answering any of them (exams were graded out of 60 points).

**Figure 5:**
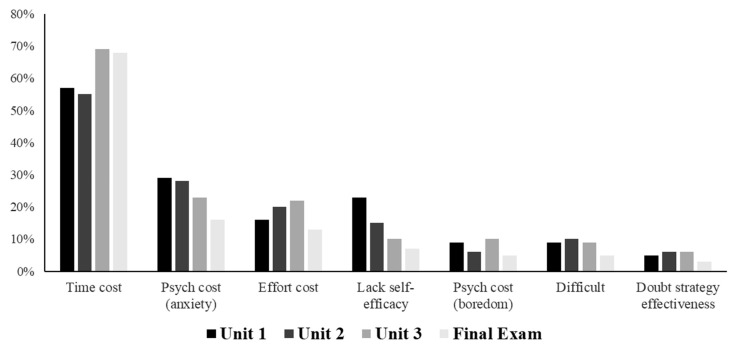
Percentage of reported barriers across unit exams.

There were five questions. First, students were asked how many points they thought they scored (out of 60) on the exam. Second, they were asked to rate how much they used “the strategies (spacing, retrieval, interleaving, generation, pretesting…) that we learned in this class to prepare for the exam,” on a scale of 1 (not at all) to 6 (fully). For this item, the strategies were referred to as a collective set; the ratings were not separated by specific strategy. Third, they were asked to rate how satisfied they were with their exam preparation, on a scale of 1 (not at all) to 6 (very satisfied). Fourth, and most central to our question of interest, students were then asked about the reasons why they did not completely use the learning strategies taught in the course, if that was the case. Seven barrier options were presented to the students, and they were instructed to circle all that applied. All seven barriers are shown in Figure 5, but for the order and full wording of each item, please see Supplemental Materials. Lastly, participants were asked to write their number one reason for not using the strategies taught in the course, either by selecting from the list presented previously or by writing their own.

### 4.2. Results

#### 4.2.1. Strategy Use and Exam Preparation Satisfaction

We examined how students’ strategy use and exam-preparation-satisfaction ratings differed throughout the exams. The means and standard deviations of the ratings by exam are presented in Table 7. The sample size for each exam varied because students either missed the exam entirely or did not complete the bonus question. We conducted two one-way within-participants ANOVAs to examine whether the average reported use of effective strategies and average reported satisfaction differed between the exams. They did not. There were no significant differences in rating of effective strategies (*F*(3, 347) = 1.66, *MSE* = 1.89, *p* = 0.176) or in how satisfied students felt in their exam preparation (*F*(3, 348) = 1.07, *MSE* = 1.39, *p* = 0.36).

#### 4.2.2. Frequency of Barriers

Figure 5 shows the percentage of students who listed each barrier across the three unit exams and the final exam. The most commonly reported barrier (61% across the four exams) to using effective learning strategies was the lack of time. As the figure shows, perceived time cost increased toward the end of the semester (rising from being mentioned by 55–57% of the students to almost 70% of the students), presumably when students had more high-stakes exams and assignments to complete. The figure also shows two other interesting trends across the semester: as the semester progressed, fewer students reported anxiety as a barrier to using the strategies, 29% reported it as a barrier in Unit 1 but only 16% reported it as a barrier on the final exam. Similarly, students became less likely to report a lack of self-efficacy as a barrier, with 23% reporting it as a barrier in Unit 1 and only 7% reporting it as a barrier on the final exam. Few students (about 5%) listed that they did not think the strategies taught in the course would be effective for them.

Chi-squared goodness-of-fit tests were conducted for each barrier, separately (see Supplemental Table S9). This relatively conservative nonparametric test showed significant differences across the semester for only one barrier: lack of self-efficacy: *χ*^2^(3) = 11.65, *p* = 0.009. This result is hopeful: it suggests that with practice, this particular barrier is likely to be resolved.

#### 4.2.3. Open-Ended Responses for Barriers

The final bonus question was an open-ended question asking participants to report the top reason why they did not use the effective learning strategies taught in the course. The sample size for the open-ended question varied per exam. Although this question was optional, the majority of students answered it: Unit 1 *n* = 87, Unit 2 *n* = 77, Unit 3 *n* = 80, and final exam *n* = 70. There were two categories coded from the open-ended responses that were not one of the options provided to students from the checklist: lack of motivation (6%; e.g., they did not feel the need to incorporate these strategies into their study) and old habits (4%; e.g., they were too anxious to try something new when their old strategies worked in the past).

Unsurprisingly, time was again the most commonly mentioned barrier, with 52% of responses mentioning time. No other reason came close to being this commonly mentioned; the second-most-common reason was anxiety (e.g., “I knew these strategies would work, but the newness to them freaked me out”), which was represented in just over 6% of the responses across the four exams. There was some variation in what people wrote about when they indicated that their barrier was lack of time. Hence, we further differentiated how these time-related responses were coded. We describe these subcategories of time barriers below, but a more detailed summary of the coding of this question is presented in Supplemental Materials (Table S10).

##### Other Responsibilities and Lack of Time to Study

The biggest share of the “no time” responses was about having other responsibilities—other classes, assignments, work. Example responses include, “I didn’t have time, because I had three other final exams today too” and “to be completely honest I also had ochem and calc exams this week and prioritized them over this one and really just ran out of time to study.” These responses represented 37% of the “no time” responses and suggest that the biggest barrier may not be about the effective strategies, per se, but more about having no time to study at all.

##### Effective Strategies Take More Time

Other responses implied that they perceived the effective strategies themselves as requiring more time. This subcategory was represented in 12% of the “no time” responses. Some of these responses implied that students believed the strategies themselves take longer compared with other strategies they could use: “I didn’t have time to use effective study strategies, so I used quick/easy strategies” and “timing was the #1 issue I faced. It was faster to go over lecture slides or notes than to do strategies we discussed in class.” Other responses implied that there are set-up costs associated with the strategies: “takes time and preparation”. A couple of times, when people mentioned their old study habits, they also mentioned that it takes time to establish new habits and new ways of studying: “I tried to apply these strategies to my own studying but it’s hard to incorporate them when you already have a way of studying. I think with practice I could be able use them more effectively” and “The times I went back to my old habits of rereading was bc my lack of time in some settings—like before a study group for a different course.”

##### Not Planning Ahead Enough to Space Study

Even though the effective strategies in the class included strategies that could be done in a single study session (e.g., retrieval practice, generation, self-explanation), about 11% of the responses in the “no time” category specifically mentioned not having planned their time far enough in advance to use spacing: “Using expanding schedules and interleaving is difficult when your schedule is always so jammed. However, this may just be out of laziness and lack of effort” and “I did not set enough time to study using the spacing strategy.”

### 4.3. Discussion

Even students who received extensive instruction on the more and less effective strategies (e.g., retrieval practice, spacing, etc.) did not always incorporate these strategies into their own studying habits. Time cost was the main barrier, and a more detailed qualitative analysis of the written responses revealed different ways that this time cost was perceived. Both anxiety about using the strategies and feeling low self-efficacy that one would be able to use these strategies effectively in their own exam preparation were not uncommon barriers. However, reports of these two barriers decreased as the semester progressed, suggesting that students can become more comfortable with using the more effective strategies.

## 5. General Discussion

Taken together, our three studies painted a nuanced picture of what students already know about effective learning and the obstacles in their way. Three major findings arose. First, in Studies 1 and 2, we showed that students can both generate, recognize, and distinguish effective strategies from less-effective strategies. Second, in Studies 2 and 3, we showed that despite having metacognitive knowledge, students also admitted to relying more on the strategies that they identified as being less effective. Third, we found that students reported several key barriers to using effective strategies. Taken together, these results imply that interventions to increase use of effective study strategies are unlikely to work if they target only metacognitive knowledge; they also need to address the perceived barriers.

### 5.1. Students Can Distinguish between More- and Less-Effective Strategies

Our undergraduate participants are more aware of the benefits of effective learning strategies than the prior cognitive psychology literature has tended to portray. In Study 1, participants were asked to imagine different levels of learners—someone at the bottom of their class, someone at the top of their class, and an average student. They not only described the top student as using all strategies more often (i.e., greater *quantity*) than the bottom and average students did, but they also described the top student as using better *quality* strategies too: the participant responses to the open-ended questions revealed many more descriptions of generative, active, and elaborative strategies among the top students, compared with the average and bottom students. The participant responses to the Likert-scale questions revealed that they understood the benefits of giving the explanations themselves instead of simply listening to them, of testing over rereading, and, more specifically, of testing throughout one’s study (both before and after studying). Similarly, in Study 2, participants shown vignettes describing different sets of learning strategies (active, passive, metacognitive) were readily able to identify the active strategies as being more likely to lead to the best learning and performance outcomes and the passive strategies as being more likely to lead to the worst learning and performance outcomes. When reporting barriers to using the active strategies, lack of efficacy (that they doubted the strategy effectiveness or did not think it would help them on their exams) was rarely the reason.

The results of Studies 1 and 2 are inconsistent with the majority of previous research, which suggests that students fail to engage in effective strategies because they do not know which strategies are effective (Bjork et al. 2013; Kornell and Bjork 2007; McCabe 2011; Yan et al. 2016); but our results are consistent with some studies that found students do have limited knowledge of certain strategies (Blasiman et al. 2017; Susser and McCabe 2013; Yan et al. 2017). Many of the studies that highlight what students do not know were published a decade ago or more. The discrepancy between the present findings and those of previous studies could reflect that students are now better informed about the effectiveness of strategies such as self-testing. Information about more-effective learning strategies is likely being shared not only via instructors but also across online and social media platforms. For example, there are increasing numbers of videos on YouTube and TikTok sharing insights on study strategies. “Learning How to Learn” is one of the most popular courses on Coursera, over 3.3 million students having been enrolled as of November 2022. Moreover, popular quizzing platforms such as Quizlet and Anki now make it easy for students to engage in retrieval practice.

There was just one strategy for which participants did not predict a difference between the different levels of students: interleaving. However, even in Study 2, their responses did not show an overwhelming preference for blocking (e.g., as in Kornell and Bjork 2008; Tauber et al. 2013; Yan et al. 2016). Rather, it seemed that they tended to split their study time between blocked study and interleaved study. This finding dovetails with earlier empirical findings by Yan and colleagues (2017), which show that hybrid schedules can be just as effective for learning, as well as with theories on how attention is differentially directed during interleaved presentation and during blocked presentation (e.g., Carvalho and Goldstone 2017). In other words, the literature shows that interleaving is not always the most beneficial strategy for learning, and students might similarly hold nuanced beliefs about the relative benefits of blocking and interleaving (see also Yan et al. 2017).

Finally, our coding of the open-ended responses to how the different imagined students reveal evidence that students are sensitive to the active processes that make strategies more or less effective. In their written descriptions of what the lower- and higher-performing students would do, they described the same strategy at differing levels of active processing (e.g., reviewing notes briefly vs. reviewing notes and trying to think up questions to ask the professor). Miyatsu et al. (2018) wrote about how even the traditionally less effective strategies (e.g., rereading, highlighting) can be made more active; it appears that our respondents share this understanding.

### 5.2. Knowledge Does Not Necessitate Usage

Students often recognize and understand the benefits of active, deeper processes and the strategies that are more likely to foster such processing. However, when directly asked about how their own study habits resembled each of the vignettes in Study 2, participants reported that their own approaches to studying for their classes more closely resembled those of the passive learner than those of the active learner. That is, students acknowledged that they use what they know to be less-effective strategies. This discrepancy between metacognitive knowledge and strategy usage is not simply due to a lack of motivation in the course: this pattern of responses was the same whether students were asked to think about how they studied for the course they cared the most about or the course they cared the least about.

While it was not always the most popular response, students often attributed struggling in class to using ineffective strategies (17–32%) and rarely attributed struggle to a lack of talent (only 6–11% of responses). However, students’ solution to struggle was rarely to change strategies (7–9%) to this problem; instead, students thought that seeking help was what was going to help those who struggle. Help-seeking is considered an adaptive response (Karabenick and Knapp 1991; Ryan and Pintrich 1997). Yet our data left it unclear as to from whom students asked for help and what kinds of advice they received. Students could be seeking help from their peers; however, previous findings have shown that peer knowledge of empirically supported strategies is limited (Morehead et al. 2016). Alternatively, students could be reaching out to their instructors or academic support centers. These sources of help are likely to have more-accurate metacognitive knowledge about how best to study but are also not immune to misconceptions about learning (Dekker et al. 2012; McCabe 2018; Morehead et al. 2016). Help-seeking has been demonstrated to be important to academic behaviors, such as metacognition, self-esteem, and strategies management (Karabenick and Knapp 1991). Therefore, future research should more carefully examine how students make decisions about *when* and from *whom* they seek help and should focus on the types of training or policies that will improve the quality of the help students receive.

### 5.3. The Barriers to Use and Potential Targets of Intervention

Taken together, the results of Studies 1 and 2 reveal that simply teaching students about effective strategies is insufficient to change their behaviors. Therefore, interventions should go beyond providing metacognitive knowledge. In Studies 2 and 3, we examined the barriers that students reported to using more-effective strategies; these barriers can reveal potential targets of intervention. We discussed four directions that could prove fruitful. The first three directions can be captured by the expectancy-value-cost framework of motivation (Barron and Hulleman 2014): increasing self-efficacy, reducing perceived time and effort cost, and reframing the more effective strategies as a more-interesting alternative to the less effective strategies. These recommendations converge with other, more-recent calls to integrate cognitive and motivational interventions (McDaniel and Einstein 2020; Zepeda et al. 2020). The fourth direction points to a way around needing intent and motivation: establishing and automating more effective study habits.

#### 5.3.1. Increasing Self-Efficacy

In both Studies 2 and 3, self-efficacy was reported as a barrier to using the more effective strategies. For example, even if a student knows that retrieval practice is good for learning, they may not know how to effectively engage in retrieval practice. Lack of self-efficacy is likely to be increased when students are not provided with resources such as practice tests to test themselves with. Of course, retrieval practice does not only take the form of practice tests or flashcards; there are many ways that retrieval practice can be engaged, e.g., free recall, covering up subheadings and trying to generate the details from memory, trying to explain a concept to someone else from memory, and so on. However, this variety of options may also be part of the reason why students do not know how to get started. In contrast, rereading is straightforward: open up the textbook or your notes and start from the first line on the first page.

Retrieval practice is often already found in the classroom. Research has found that middle school teachers frequently (almost 50% of the time) ask students questions in a way that gives students an opportunity for retrieval (Fazio 2019). Teachers might also use bell ringers (having students answer questions at the beginning of class), pose questions and require clicker responses or think-pair-share activities throughout the class, and use exit tickets and reflections at the end of class to prompt active processing and retrieval from memory. However, students might not recognize these as retrieval practice activities if attention is not drawn explicitly to them. Alternatively, students might think that retrieval practice is useful for knowing what they do and do not know, rather than being activities that directly strengthen learning (Carpenter et al. 2020; Rivers 2021). Interventions therefore might explore the benefits of having teachers more explicitly explain the pedagogical benefits of the activities and structures of their courses and the benefits of giving students more practice in engaging more-effective strategies in the classroom. These interventions might increase students’ self-efficacy in using these more effective strategies in their self-regulated learning. Indeed, the participants in Study 3 were enrolled in a course that involved generative classroom activities and spaced and interleaved retrieval practice features; as the semester progressed, students became less likely to report a lack of self-efficacy as a barrier.

#### 5.3.2. Reducing Perceived Costs

The most commonly reported barriers to using the more effective strategies were perceived time and effort costs. The two types of costs are related: students might believe that using more-effortful strategies means that they will have to put in more time. The empirical evidence, however, shows that the more effective strategies do not have to take more time; they make study more efficient. Most of the experiments that have compared more- and less-effective strategies, for example, controlled for total study time. Perhaps this message is not being conveyed well enough to students.

However, there are other ways that these more effective strategies might legitimately take more time. Open-ended responses in Study 3 revealed that participants often left their studying too late to effectively engage spacing. In other words, even though total study time might be the same, spacing does still require students to start sooner. Retrieval practice might also feel like it takes more time because one has to first decide *how* to engage in retrieval practice (e.g., does the student need to make or find practice questions and flashcards?). It could be that simply having more practice with these strategies would be enough to address the perceived time and effort costs. For example, giving students practice with free recall as a retrieval practice technique could show them that it does not require any additional preparation time and can in fact better direct their study because it quickly shows them what they do and do not know. The point is that there are ways around these perceived barriers, but interventions need to make an explicit effort to get around them.

#### 5.3.3. Reframing More-Effective Strategies as More Interesting

The most common barrier to using the passive strategies was that students found studying with those strategies boring. If these passive strategies are indeed students’ habitual approaches to study, then that might explain why so many students find school boring (Busteed 2013; Lyons 2004). However, active and metacognitive strategies were rarely described as boring. We propose that both active and metacognitive strategies can be encouraged by reframing them as ways to make studying not only more effective but also more *interesting*. Rather than have students focus on the fact that more-effective strategies require more effort, messaging might have students focus on how these strategies can make the act of studying more engaging. Some evidence in support of this possibility comes from classroom studies showing that the incorporation of more-active and -elaborative teaching practices is related to increased student engagement and satisfaction (Muenks et al. 2021; Roach 2014; Unal and Unal 2017). In this way, self-regulated learning interventions might resemble those of stress reappraisal interventions (Brady et al. 2018; Liu et al. 2019), reducing the perceived costs of engaging more-effective strategies and increasing perceived benefits beyond learning.

#### 5.3.4. Establishing New Study Habits

Finally, one theme that our coding of the open-ended responses in Study 3 revealed is that when time was running low, students sometimes reported that this feeling of urgency led them to fall back on their old study habits. When time feels short, it can be anxiety provoking to try new ways of studying, especially if one’s old ways of studying had been successful enough to get them to university. Habits are context-dependent, automatically initiated behaviors. Habits can be overridden with conscious effort, but when people feel rushed (e.g., because time is running low) or tired, they are more likely to fall back on old habits (Wood and Rünger 2015). The problem, however, is that in college, students are often stressed and low on time (van der Meer et al. 2010) and find their study decisions being driven by the next most-pressing deadline (Kornell and Bjork 2007). Moreover, by the time students reach college, they already have well-established habits, and these habits might involve a lot of less-effective strategy use. Interventions therefore might focus on helping students establish more-effective study habits, but a single-session intervention is likely to be insufficient. The benefit, however, of creating effective habits is that once a habit has been formed, contextual cues are often sufficient to initiate behavior and motivation is no longer needed (Wood and Rünger 2015; see also Fiorella 2020).

### 5.4. Limitations of the Present Studies

One limitation of Studies 1 and 2 is that we did not collect information about whether our participants had been directly taught these strategies. In other words, we do not know whether our data imply that existing training on study strategies is relatively effective at teaching students what is or what is not effective (and whether this training comes from more-formal sources in school or from less-formal sources, such as social media) or whether our data imply that this is knowledge that students are able to intuit naturally.

Another limitation of Study 2 is that the vignettes were not presented in a randomized order. Student A was always the active learner; Student B was always the passive learner; and Student C was always the metacognitive learner. Hence, responses could have been influenced by order effects. We believe that the response patterns, however, would largely look the same even if the vignette order had been randomized. For example, participants did not simply decide that the order of presentation was equivalent to the order of strategy efficacy, as the learning and the performance of Student C were rated as falling in between those of Students A and those of B, respectively. Moreover, participants did not first answer every single question about Student A, followed by all the questions about Student B and then Student C. Rather, for each type of question, participants provided their responses to all three student vignettes. In other words, if participants were fatigued by the end of the study, that should have affected their responses to all three vignettes, not just vignette C. Additionally, we acknowledge that the three vignettes do not always represent entirely different strategies. In particular, it is possible that participants interpreted the active strategies as subsuming those of the other two sets of strategies; for example, spacing out study might also involve planning study (a strategy used by the metacognitive student), and quizzing oneself might be followed up with rereading (a strategy used by the passive student).

Finally, in Studies 2 and 3, participants were undergraduates enrolled in a public research-intensive four-year university in the United States. Therefore, they should not be considered representative of all students. However, they do resemble the type of student population that the earlier research has drawn from. Thus, they might be considered comparable to the students in the studies that conclude that students do not have metacognitive awareness of effective learning strategies. In Study 1, however, we recruited self-identified students from a much broader participant pool, Prolific. Although we did not collect information about what kind of college participants were enrolled in, it was unlikely that they all came from research-intensive four-year universities. Hence, Study 1 provides some evidence for the generalizability of metacognitive knowledge, at least across US undergraduate students.

### 5.5. Concluding Comments

Our present studies highlighted the nuances with which students understand more- and less-effective strategies for study. Taken together, our data painted an optimistic picture that whatever combinations of sources students were gaining knowledge from, such as formal education, informal sources, or personal experiences, they did seem to be aware of the benefits of actively processing information. However, our data also pointed to the need for a lot more work on understanding how effective interventions can be designed. We provided both suggestions for motivational interventions (that addressed self-efficacy, perceived costs, and otherwise motivate the benefits of using these strategies) and interventions that focused on establishing better study habits (and hence circumvent the need for motivation).

## Figures and Tables

**Table 1:** Likely Reasons for Academic Struggles.

Reason for Struggle	Bottom 10%	Average	Top 10%
Using ineffective strategies	27 (25%)	33 (32%)	16 (17%)
Lack of preparation	9 (8%)	12 (12%)	17 (18%)
Teacher	2 (2%)	9 (9%)	23 (25%)
Lack of effort	25 (24%)	17 (17%)	2 (2%)
Lack of talent	7 (7%)	6 (6%)	10 (11%)
Distracted	36 (34%)	25 (25%)	24 (26%)

**Table 2:** Likely Behaviors in Response to Struggle.

Behavioral Response to Struggle	Bottom 10%	Average	Top 10%
More study time	14 (13%)	18 (18%)	19 (21%)
Seek help	42 (40%)	49 (48%)	51 (55%)
Self-learn from other resources	10 (9%)	17 (17%)	14 (15%)
Change study strategies	7 (7%)	9 (9%)	6 (7%)
Give up	33 (31%)	9 (9%)	2 (2%)

**Table 3:** Use of Testing: Frequency and Percentage of Responses by Condition.

Item	Bottom 10%	Average	Top 10%
a. Review everything first and then test themselves on all the content	69 (65%)	28 (27%)	10 (11%)
b. Testing themselves on everything first, reviewing, and then testing themselves on everything again	13 (12%)	31 (30%)	25 (27%)
c. Reviewing content in chunks, testing themselves after each chunk	16 (15%)	24 (24%)	21 (23%)
d. Splitting content into chunks, testing themselves before and after reviewing each chunk	8 (7.5%)	19 (19%)	36 (39%)
Dimension	Bottom 10%	Average	Top 10%
Chunking (vs. everything at once)	24 (23%)	43 (42%)	57 (62%)
Testing before and after (vs. only after)	21 (20%)	50 (49%)	61 (66%)

*Note*: The percentages shown in the parentheses reflect the percentage of responses within each condition. The chunking dimension reflects the summation of items c and d. The testing before and after each dimension reflects the summation of items b and d.

**Table 4:** Qualitative Coding Categorizing Learning Strategies by Condition.

Code	Overall	Bottom 10%	Average	Top 10%	Strategy Type
Reviewing or rereading	77%	76%	75%	79%	Passive
Flashcards or self-test	34%	15%	41%	47%	Active/Passive
Practice problems	21%	6%	29%	29%	Active
Create study aids	18%	8%	18%	29%	Active
Study group	14%	8%	18%	16%	Active/Passive
Metacognition	13%	8%	15%	18%	Active
Use online resources	12%	14%	9%	13%	Active/Passive
Seek help	10%	6%	12%	14%	Active/Passive
Memorization	9%	11%	7%	8%	Passive
Space out learning	8%	1%	9%	16%	Active
Skim	8%	22%	1%	0%	Passive
Cram	6%	11%	5%	0%	Passive
Explain to self or others	4%	1%	4%	9%	Active
Highlight/underline	3%	1%	3%	4%	Passive
Not study	3%	8%	0%	0%	Passive

*Note*: The “strategy type” category refers to whether the strategy is likely to encourage more-active processing; each strategy, however, can be used in ways that are more or less active.

**Table 6:** Categorical Responses to Which Student Would Learn and Perform the Best in Study 2.

	Active Student	Passive Student	Metacognitive Student	*χ*^2^(2)
Performs best on the exam	406 (79%)	38 (7%)	73 (14%)	478.80 ***
Uses the most effective strategies	361 (70%)	38 (7%)	118 (23%)	328.39 ***
Learns the most	312 (60%)	51 (10%)	154 (30%)	200.57 ***
Remembers the most a year from now	435 (84%)	30 (6%)	52 (10%)	601.93 ***

*Note*: *** *p* < 0.001.

**Table 7:** Means and Standard Deviation of Strategy Use and Satisfaction Ratings by Exam.

	Unit 1 *n* = 88	Unit 2 *n* = 89	Unit 3 *n* = 86	Final Exam *n* = 92
Strategy Use	3.90 (1.01)	4.16 (0.93)	3.65 (1.11)	3.83 (1.18)
Satisfaction	3.73 (1.07)	4.02 (1.11)	3.69 (1.18)	3.59 (1.19)

*Note.* Each item was rated on a 6-point scale, where 1 = not at all and 6 = completely/very satisfied.

## Data Availability

The materials and data presented in this study are openly available in Open Science Framework at https://osf.io/4d9e3/ (made available 9 September 2022).

## Note

1 In general, the better the student, the more they were described as being positively motivated (higher value, higher achievement goals, higher in difficulty-as-importance, lower in difficulty-as-impossibility, and lower in failure-as-debilitating beliefs). There were no differences, however, in the learning-should-feel-easy belief.

## Supplementary Materials

The supplementary materials can be found at https://osf.io/4d9e3/ (uploaded 9 September 2022). Table S1: Frequency of Learning Strategy Use: Descriptive Statistics and Effect Size of Differences Between Conditions in Study 1. Table S2: Use of Testing by Condition in Study 1, Chi-square Goodness-of-Fit Results. Table S3: Quality of Strategy Use: Mean (and Standard Deviation) Percentage of Time Using the More (vs. Less) Effective Strategy in Study 1. Table S4: Wording of the Vignettes in Study 2. Table S5: MANOVA Results Between Vignettes and Performance and Learning Ratings. Table S6: Descriptives for Ratings on Performance and Learning per Vignette. Table S7: Descriptives for Ratings on Strategies Similarities. Table S8: ANOVA and Pairwise Comparisons: Similarity of Vignette Strategies to Own Study Behavior. Table S9: Chi-square Goodness-of-fit Tests: Did the Reported Barriers Differ Across Exams? Table S10: Coding of Open-Ended Responses for Top Barrier to Using Effective Strategies.
